# Association of ovarian stimulation and embryonic aneuploidy in *in vitro* fertilization cycles with preimplantation genetic testing: A narrative systematic review

**DOI:** 10.5935/1518-0557.20210069

**Published:** 2022

**Authors:** Jorge Rodriguez-Purata, Maria Jose Gomez-Cuesta, Enrique Cervantes-Bravo

**Affiliations:** 1 Clinica de la Fertilidad "CdelaF", Prolongacion Vasco de Quiroga 4001, Torre A Piso 10, Colonia Santa Fe, Cuajimalpa de Morelos, 05370, Mexico City, Mexico; 2 Centro Medico ABC, Avenida Carlos Graef Fernández 154, Colonia Santa Fe, Cuajimalpa de Morelos, 05300, Mexico City, Mexico

**Keywords:** aneuploidy, *in vitro* fertilization, assisted reproductive techniques, ovarian stimulation, preimplantation diagnosis, preimplantation genetic testing

## Abstract

The impact of gonadotropins used for COS on the rate of embryo aneuploidy in patients without the negative effects of age as a confounding factor, is still a subject of lively debate. We ran a systematic search for studies in MEDLINE, PubMed, Google Scholar and the Cochrane Library. A librarian coordinated the search in December of 2020. We included all original peer-reviewed papers in English, irrespective of study-design. There were no restrictions concerning method of amplification or platform used to analyze the amplified DNA. We used the PICO model to select the study population. We included women/couples submitted to COS for IVF with the intention to genetically analyze her/their embryos through PGT. The primary outcome was the rate of aneuploidy. We used the Newcastle-Ottawa scale (NOS) score to evaluate the quality of the studies included. The search yielded 73 citations, and 14 were eligible for analysis, which included data on 4805 cycles. Media quality NOS score was 8. Although it has been demonstrated that natural cycles are associated with aneuploidy, it does seem that more robust stimulations are indeed associated with a higher proportion of aneuploidy. Nevertheless, a higher response is associated with an increased number of euploid embryos available for transfer, which translates into more embryo-transfer cycles with a prospective higher cumulative live birth rate. Further evidence is needed to ascertain if there is a negative impact of COS, especially at the cellular level.

## INTRODUCTION

Since the pioneering days of *in vitro* fertilization (IVF) (Trounson *et al*., 1981), controlled ovarian stimulation (COS) has been an integral part of assisted reproductive technology (ART) treatment. Primarily, the goal of COS is to induce ongoing development of multiple dominant follicles in a single cycle and to mature as many oocytes possible to improve chances for conception either in vivo (empirical ovarian stimulation with or without intrauterine insemination) or *in vitro* (with IVF) (Macklon *et al*., 2006). Although several advantages have been thoroughly described (Macklon *et al*., 2006; Drakopoulos *et al*., 2016; Rodriguez-Purata *et al*., 2016), data is still missing regarding the association of COS and the incidence of embryonic aneuploidy.

Commensurate with the developments in COS (Macklon *et al*., 2006), the more recent implementation of preimplantation genetic testing (PGT) of embryos introduced by Handyside *et al*. (1989) into the available strategies for treating infertile patients, is allowing the checking of all 24 chromosomes within an embryo before transfer. To date, several improvements have been made to the technology (Scott *et al*., 2013; Sermon *et al*., 2016) that have enhanced its validity and propelled its adoption (De Rycke *et al*., 2015). But even with these improvements, its true value still remains unknown (Practice Committees of the American Society for Reproductive Medicine and the Society for Assisted Reproductive Technology, 2018; Munné *et al*., 2019).

As a result of the improvements made to the technology, more information is recently available regarding the impact of COS on embryonic aneuploidy. This systematic review assesses the current evidence presented on critical clinical questions concerning the association of COS in women undergoing IVF coupled with PGT and embryonic aneuploidy.

### Origin and Mechanisms of Embryonic Aneuploidy in Humans

One of the main concerns is that exogenous follicle stimulating hormone (FSH) may be correlated with an increased risk of embryonic aneuploidy (Elbling & Colot, 1985; Sato & Marrs, 1986; Edgar *et al*., 1987). Whereas animal studies seem to support an association (Santaló *et al*., 1986), human studies have been more conflicting (Baart *et al*., 2007; Verpoest *et al*., 2008; Rubio *et al*., 2010; Kyrou *et al*., 2011; Massie *et al*., 2011; Ata *et al*., 2012; Labarta *et al*., 2012; 2017; Barash *et al*., 2017; Sekhon *et al*., 2017; Venetis *et al*., 2019).

Almost all recent information on the chromosomal status of the early human embryo has been derived from the outcome of PGT and follow-up studies thereof. Overall, ART has helped us learn so much about aneuploidy (Nagaoka *et al*., 2012; Rodriguez-Purata *et al*., 2015), but it makes us think it is even more prevalent. Before ART, knowledge came essentially from newborns, stillbirths, and abortions (Nagaoka *et al*., 2012). Nowadays, ART provides data of the stage at which segregation errors become evident, from female meiosis to the blastocyst stage of preimplantation embryo development (Capalbo *et al*., 2013).

Due to the prevalence and significance of aneuploidy in humans, it is important to understand the origins and mechanisms of development. For a more in-depth analysis, please refer to (Delhanty, 2005; Fragouli *et al*., 2013; Taylor *et al*., 2014).

### Possible Mechanisms for Aneuploidy by COS

The effect of high doses of gonadotropins is not yet well-understood, especially at the most basic level of organization - the cellular level. In theory, it has been speculated that high doses of exogenous gonadotropins administered over a prolonged interval interfere with natural selection, generating FSH-induced chromosome dysfunction in oocytes or recruitment of poor-quality oocytes when the natural selection process of a dominant follicle is superseded by COS (Nagaoka *et al*., 2012).

#### Evidence from non-human models

COS influence has been well-characterized in animals, mainly in rodents, and has shown that intense COS could lead to poorer embryonic development potential, and could increase the rate of chromosomal defects. Mice exposed to high doses of gonadotropins had reduced blastulation, a higher rate of chromosomal aberrations, and increased embryonic degeneration (Vogel & Spielmann, 1992). In contrast, other animal studies have shown a lack of difference in the incidence of nondisjunction in mice oocytes obtained after COS versus natural cycles (Golbus, 1981). More recently, experimental evidence in mice showed that alterations in the multiple signals that regulate folliculogenesis could change the later stages of oocyte development, increasing the risk of poor chromosomal segregation in subsequent meiotic divisions (Hodges *et al*., 2002).

#### Evidence from human studies

Unfortunately, human studies to date bear some degree of conflicting results and conflicting conclusions, possibly due to significant differences in study design and the technology used to assess ploidy. In general, COS has been proposed to influence oocyte maturation and completion of meiosis, potentially mediating chromosomal aneuploidy and mosaicism (Verberg *et al*., 2009). Additionally, elevated serum estradiol levels and oocyte yield have been associated with increased blastomere multinucleation (Jackson *et al*., 1998).

Recent observations suggest that inaccuracies in the chromosomal segregation mechanism in oocytes are often involved, and this process is triggered by maternal age (Hassold & Hunt, 2001; Champion & Hawley, 2002). Inaccuracies in chromosome segregation in oocytes may be responsible for embryonic aneuploidy, and may be induced by failures in the mechanisms that control cell cycle, thus affecting the entire chromosome segregation process (Barlow & Hultén, 1998; Tease *et al*., 2002); whereas segregation of particularly unstable bivalents responsible for aneuploidies arising during the first meiotic division can be caused by synaptic errors between specific homologous chromosomes, or a reduction in the number of chiasms due to a low recombination rate (Nicolaidis & Petersen, 1998; Hassold & Hunt, 2001; Champion & Hawley, 2002; Obradors *et al*., 2010). The COS used for IVF could be responsible for these abnormalities in chromosome segregation; hence the presence of aneuploidy and mosaicism in embryos due to changes to the cytoskeleton and/or the mitotic spindle in the first embryonic division of bipronuclear zygotes (Munne *et al*., 1997).

Furthermore, analysis of human gametes revealed that embryo aneuploidy is caused primarily by an error-prone meiotic chromosomal segregation mechanism in oocytes. If COS can exert any deleterious effect on the embryo, this may be because it induces meiotic errors in the oocyte (a uniformly aneuploid monosomal, trisomic, haploid, or polyploid embryo), but not mitotic errors after fertilization (mosaicism) (Frumkin *et al*., 2008). At the genomic level, the increased incidence of genomic imprinting disorders in oocytes after ovulation induction and in vitro maturation may be due not only to maternal factors such as age, but also to external factors such as the biochemistry of the abnormal or suboptimal follicular fluid and cytoplasmic development (Sato *et al*., 2007).

## MATERIALS AND METHODS

### Protocol and Registration

The study complied with the Preferred Reporting Items for Systematic Reviews and Meta-Analyses (PRISMA) guidelines (Moher *et al*., 2009). The study protocol was registered at http://www.crd.york.ac.uk/PROSPERO/ (CRD42019120803) before starting the review. This study was exempted from the institutional review board approval, because it is a systematic review.

### Eligibility Criteria

We used the PICO (Patients, Intervention, Comparison and Outcomes) model to select the study population (Schardt *et al*., 2007), and we included women/couples who underwent COS for an IVF cycle with the intention to genetically analyze her/their embryos through PGT.

### Search Strategy

The authors performed a systematic search of the literature for studies appropriate to the clinical question on the Medical Literature Analysis and Retrieval System Online (MEDLINE), ClinicalTrials.gov, PubMed, Google Scholar, and the Cochrane Library (http://www.cochranelibrary.com). We included a combination of MeSH terms and/or text words relevant to the association of COS with embryonic aneuploidy in the setting of an IVF cycle with PGT in the search builder (Supplemental Table 1). The authors additionally checked citations on the Web of Science and manually searched the references of the papers. Searches were coordinated by an expert librarian and a statistician in September'20. Search updates were conducted in December'20. All records were screened for eligibility by two independent reviewers. We included all original English, peer-reviewed articles, irrespective of study-design, were included.

### Study Selection

Two reviewers (J.R-P./E.C-B) independently evaluated titles and abstracts. Duplications were removed using the Zotero online software and manually, when needed. Final inclusion/exclusion decisions were made upon examining the papers in full. We resolved discrepancies by discussion and consensus between authors, with the involvement of another author (MJ.G-C). To acquire the highest level of evidence, we selected randomized trials and observational studies, including cohort- and case-control studies. Trials published only as abstracts, letter to the editor, editorials, or studies retracted from the literature after their publication were excluded upfront. Systematic reviews were also excluded, but we checked the references first.

Intervention studies were eligible if: 1) randomized controlled trials (RCTs), non-RCTs, prospective observational studies, or cohort studies; 2) assessed the impact of COS on generating aneuploid embryos and/or conceptions; 3) under any type of stimulation protocol (long agonist, antagonist, microflare, estrogen priming, progesterone-primed, etc.) comparing the study group *vs*. no COS or COS with a reduced dose; and 4) collected the status of the biopsied embryos as euploid/aneuploid endpoint. There were no restrictions in terms of amplification method, or the platform used to analyze the amplified DNA.

### DATA Extraction and Quality Assessment

Eligible records were systematically collected and imported into an electronic database by two reviewers (J.R-P. and E.C-B), using predefined data fields and collected in a standardized data extraction form. This included the following data: year of publication, study design, study period, intervention, number of patients (total, exposed and unexposed), PGT platform, biopsy day (cleavage or blastocyst stage biopsy), number of cells biopsied, age criteria, general inclusion criteria, control and study groups, average of oocytes retrieved, total number of euploid embryos, euploid/aneuploid ratio. Additionally, for intervention studies, allocation concealment and blinding were also recorded. In case of multiple publications we collected, the most up-to-date or comprehensive information.

The studies included were further subclassified according to the stimulation protocol: COS *vs*. no COS; low *vs*. conventional dose; COS under a long-agonist *vs*. antagonist; FSH-only *vs*. luteinizing hormone (LH)-containing gonadotropins; trigger medication used; and according to ovarian response. Within subgroups, special attention was given to oocytes retrieved, total number and average of euploid embryos, and proportion of euploid/aneuploid embryos.

When specifically comparing COS *vs*. no COS and conventional *vs*. reduced dose, we ran an intention-to-treat (ITT) analysis (Sedgwick, 2015) in order to provide a comparable analysis between groups and specifically answer the following question: if a patient decides to undergo a PGT cycle, which strategy provides the higher odds of developing a euploid embryo? ITT analysis means all patients who were enrolled and randomly allocated to treatment were included in the analysis and were analyzed in the groups to which they were initially randomized. This analysis is particularly relevant in this review, given that it has been previously established that patients who do not reach retrieval or biopsy stage are potentially aneuploid oocytes/embryos (Qi *et al*., 2014).

### Appraisal of Certainty of Evidence

Two authors (J.R-P. /E.C-B.) independently assessed the risk bias of each study. We used the Newcastle-Ottawa scale (NOS) to evaluate the case-control or cohort studies included, and judgment on each one was passed according to three issues: selection of the study group, comparability between groups and the ascertainment of outcomes and exposures of interest (Hartling *et al*., 2012). This scale gives up to 9 stars to each study and classifies them as low quality (0-4 stars), moderate quality (5-6 stars) and high quality (7-9 stars) (Wells *et al*., 2014) (Supplemental Table 2).

The evaluation was performed independently by two reviewers (J.R-P., E.C-B), while any disagreement was resolved by discussion between the two parties or by including a third reviewer (MJ.G-C.).

We did not run a meta-analysis because of the large heterogeneity in the definitions of COS exposure, biopsy stage, and platform used.

## RESULTS

### Study Selection

The literature search retrieved 74 citations, of which 11 were eligible for further consideration after screening titles and abstracts. Cross-reference checking resulted in an additional inclusion of four papers. Haaf *et al*. (2009) was not detected because it does not include any of the Medical Subject Headings (MeSH) terms or text words related to IVF. Rubio *et al*. (2010) and Thorne et al. (2019) were not detected because they did not include any of the MeSH terms or text words related to the PGT procedure, type of analysis or type of platform. Ata et al. (2012) was not detected because it does not include any of the MeSH terms or text words related to COS (Figure 1).

### Description of Studies Included

Overall, 15 studies published before December 2019 fulfilled our inclusion criteria, and included data on 5344 cycles (Baart *et al*., 2007; Verpoest *et al*., 2008; Weghofer *et al*., 2008; 2009; Haaf *et al*., 2009; Rubio *et al*., 2010; Kyrou *et al*., 2011; Massie *et al*., 2011; Ata *et al*., 2012; Labarta *et al*., 2012; 2017; Barash *et al*., 2017; Sekhon *et al*., 2017; Thorne *et al*., 2019; Venetis *et al*., 2019) were included (Table 1). There are five studies from the USA, three from Spain, two from Belgium and Austria/USA, respectively, and one from Australia, Germany and the Netherlands, respectively. There is one RCT (Baart *et al*., 2007), four prospective non-randomized trials (Verpoest *et al*., 2008; Rubio *et al*., 2010; Labarta *et al*., 2012; 2017) and ten retrospective studies (Weghofer *et al*., 2008; 2009; Haaf *et al*., 2009; Kyrou *et al*., 2011; Massie *et al*., 2011; Ata *et al*., 2012; Barash *et al*., 2017; Sekhon *et al*., 2017; Thorne *et al*., 2019; Venetis *et al*., 2019). In terms of platform used, three studies used array comparative genomic hybridization (aCGH) (Ata *et al*., 2012; Thorne *et al*., 2019; Venetis *et al*., 2019), one study used quantitative polymerase chain reaction-based comprehensive chromosome screening (qPCR-based CCS) (Sekhon *et al*., 2017), one study used single nucleotide polymorphism (SNPs) (Barash *et al*., 2017), and ten underwent fluorescence in situ hybridization (FISH) analysis. In terms of biopsy stage, three studies included patients whose embryos underwent biopsy at the blastocyst stage (Barash *et al*., 2017; Sekhon *et al*., 2017; Thorne *et al*., 2019), nine studies included patients whose embryos underwent biopsy at the cleavage stage, and one study included both stages, although analyzed independently (Ata *et al*., 2012). Lastly, two studies providing an indirect measurement of embryonic aneuploidy in relation to COS exposure: one study analyzed polar bodies (Haaf *et al*., 2009) and one study analyzed products of conception (POC) (Massie *et al*., 2011).

**Table 1.** Characteristics of the studies included.

Author	Country	Study Period	Design	Intervention	Control Group	Study Group	
Baart *et al*., 2007	Netherlands	Dec'02-Aug'05	RCT	Low *vs*. Conventional Stimulation	150 IU / antagonist	225 IU / long agonist	
Weghofer *et al*., 2008	Austria/USA	Jan'04-Jun'05	Retrospective	FSH *vs*. LH-containing gonadotropins/agonist protocol	FSH	hMG	
Verpoest *et al*., 2008	Belgium	Oct'05-Apr'06	Prospective observational study	Aneuploidy in Unstimulated Cycles	No control group	Unstimulated cycles	
Haaf *et al*., 2009	Germany	Jan'02-Dec'05	Retrospective	Oocyte aneuploidy by age and response (1-5, 6-10, or >10 oocytes)	<35 years	35-40 years; >40 years	
Weghofer *et al*., 2009	Austria/USA	Jan'03-Jun'05	Retrospective	FSH *vs*. LH-containing gonadotropins/antagonist protocol	FSH	hMG	
Rubio *et al*., 2010	Spain	NA	Crossover study	1st cycle normal dose *vs*. 2nd cycle 30% reduce dose, 3 months apart	225 IU/long agonist	150 UI/antagonist	
Kyrou *et al*., 2011	Belgium	Oct'92-Dic'06	Retrospective	GnRH agonist *vs*. GnRH Antagonist	Agonist	Antagonist	
Massie *et al*., 2011	USA	Jan'99-Aug'07	Retrospective	Aneuploidy in miscarriages from stimulation *vs*. no stimulation	Natural conceptions	Stimulation	
Ata *et al*., 2012	USA	Jan'10-Jul'11	Retrospective	Aneuploidy according to embryos biopsied	NA	NA	
Labarta *et al*., 2012	Spain	Sep'06-Mar'10	Prospective crossover study	1st cycle unstimulated *vs*. 2nd cycle stimulated, same patient	Unstimulated	Stimulated with 225 IU	
Sekhon *et al*., 2017	USA	Mar'10-Apr'15	Retrospective	Cumulative dose and aneuploidy rate	NA	NA	
Labarta *et al*., 2017	Spain	Sep'06-Mar'10	Post hoc analysis of prospective crossover study	Aneuploidy according to ovarian response (same-dosage): low *vs*. high	Low: <17 oocytes	High: ≥17 oocytes	
Barash *et al*., 2017	USA	Jan'13-Jan'13	Retrospective	Different dosages of gonadotropins and aneuploidy rate	NA	NA	
Venetis *et al*., 2019	Australia	Mar’11-Dec’16	Retrospective, Multicenter	Correlation of oocytes retrieved and the number of euploid embryos	NA	NA	
Thorne *et al*., 2019	USA	Jan’13-Feb’18	Retrospective	Final oocyte maturation trigger rhCG *vs*. aGnRH	rhCG trigger	aGnRH trigger	
**Author**	**Inclusion Criteria**	**Patients**	**Patients by Group**	**PGT platform**	**Biopsy Day**	**Cells Biopsied**	**Cells Biopsied**
Baart *et al*., 2007	General IVF patients, regular menses, 5 mill sperm, 46XX	111	Low=67 High=44	FISH (10 chromosomes)	Day 3	Cells Biopsied	Low=33.2 High=34.1
Weghofer *et al*., 2008	General IVF patients, regular menses, normal ovarian function, 46XX	104	FSH=52 hMG=52	FISH (9 chromosomes)	Day 3	1-2 cells	FSH=35.0 hMG=35.9
Verpoest *et al*., 2008	Regular menses, <3 failed cycles, normal uterine cavity	30	Unstimulated=30	FISH (5 chromosomes)	Day 3	1 cell	31.4
Haaf *et al*., 2009	>35 years or previous IVF cycle with no ET	30	<35: 93 cycles 35-40: 376 cycles >40: 165 cycles	FISH (5 chromosomes)	Day 0	1 cell	NA
Weghofer *et al*., 2009	General IVF patients, regular menses, normal ovarian function, 46XX	104	FSH=52, hMG=52	FISH (9 chromosomes)	Day 3	2 cells	FSH=39.8 hMG=39.6
Rubio *et al*., 2010	Oocyte donors	32	First cycle= 32 Second cycle=22 (10 no biopsy)	FISH (9 chromosomes)	Day 3	1 cell	22.0
Kyrou *et al*., 2011	Recurrent Miscarriage, RIF, azoospermia	694	Agonist=320 Antagonist=374	FISH (7 Cells Biopsied)	Day 3	1-2 cells	Agonist=32.8 Antagonist=32.7
Massie *et al*., 2011	Infertility patients trying to conceive	229	No stimulation=50 FSH for IUI=35 FSH for IVF=144	Trypsin-Wright G-banding	NA	1 cell	Natural 37.0 FSH exposure 37.0
Ata *et al*., 2012	Infertility patients undergoing PGT or Oocyte donors	990	7753 embryos	aCGH-based CCS	Day 3/Day 5	POCs	NA
Labarta *et al*., 2012	Oocyte donors	232	Unstimulated=185 Stimulated=47	FISH (9 chromosomes)	Day 3	Day 3: 1-2 cells; Day 5: 3-10 cells	25.4
Sekhon *et al*., 2017	Infertility patients undergoing PGT	828	1122 cycles	qPCR-based CCS	Day 5	1 cell	38.5
Labarta *et al*., 2017	Oocyte donors	46	Low response=22 High response=24	FISH (9 chromosomes)	Day 3	4-7 cells	Low=25.6 High=24.9
Barash *et al*., 2017	Infertility patients undergoing PGT	681	794 cycles	SNP	Day 5	4-9 cells	NA
Venetis *et al*., 2019	Infertility patients undergoing PGT	724	724 cycles	aCGH	Day 3	1 cell	NA
Thorne *et al*., 2019	Infertility patients undergoing PGT	539	hCG trigger=336 aGnRH trigger=203	aCGH/NGS	Day 5	NA	No restriction
**Author**	**Primary Outcome measure**	**Oocytes retrieved**	**Aneuploidy Rate per embryo**	**Aneuploidy Rate by ITT**	**Euploid Embryos (n)**	**Euploid Embryos by ITT (n)**	
Baart *et al*., 2007	Oocytes retrieved Aneuploidy Rate	Low=8.2 High=12.1	Low=50% High=62%, *p*=0.48	Low=22% High=26%, *p*=0.4	Low=1.8 High=1.8	Low=1.4 High=2.5	
Weghofer *et al*., 2008	Oocytes retrieved Aneuploidy Rate	FSH=23.7 hMG=20.3	FSH=54.7% hMG=30.2%, *p*<0.01	Only patients with biopsy included	FSH=3.1 hMG=3.3	NA	
Verpoest *et al*., 2008	Aneuploidy Rate	Unstimulated =0.6	Unstimulated=36.4%	NA	Unstimulated =0.2	NA	
Haaf *et al*., 2009	Aneuploidy per cohort size (1-5,6-10, >10 oocytes) and per age (<35, 35–40, >40)	<35: 8.5 35-40: 8.6 >40: 7.1	1-5 oocytes (<35: 23.3%; 35-40:41.2%; >40:49.6%) 6-10 oocytes (<35:34.9%; 35-40: 43.8%; >40: 50.0%) >10 oocytes (<35:50.9%; 35-40:54.6%; >40:54.1%)	NA	NA	NA	
Weghofer *et al*., 2009	Oocytes retrieved Aneuploidy Rate	FSH=15.2 hMG=13.8	FSH=70.6% hMG=74.3%, NS	NA (only patients with biopsy included)	FSH=2.1 hMG=1.9	NA	
Rubio *et al*., 2010	Oocytes retrieved Aneuploidy Rate	Standard=23.9 Reduced=14.7	Standard=83.9% Reduced=77.5%	NA	Standard=3.1 Reduced=2.7	Standard=3.1 Reduced=1.8	
Kyrou *et al*., 2011	Oocytes retrieved Aneuploidy Rate	Agonist=12.9 Antagonist=13.6	Agonist=49.9% Antagonist 50.2%, *p*=0.922	NA (only patients with biopsy included)	Agonist=2.7 Antagonist=3.1	NA	
Massie *et al*., 2011	Aneuploid gestation	NA	Natural=70% FSH exposure=63%, NS	NA	NA	NA	
Ata *et al*., 2012	Aneuploidy per cohort size (1-4, 5-7, 8-10, >10 embryos) and per age (<35, 35–39, 40–42, ≤43)	NA	Per age and (D3/Blastocyst): <35 (38.3%/61.4%) 35–39 (28.9%/50.27%) 40–42 (16.05% /33.37%); ≤43 (8.95%/17.3%)	NA	NA	NA	
Labarta *et al*., 2012	Aneuploidy Rate	Unstimulated =0.8 Stimulated=17.8	Unstimulated=35.3% *vs*. Stimulated=40.6%	Unstimulated =76.7% Stimulated =40.6%	Unstimulated =1.8 Stimulated=3.9	Unstimulated =1.0 Stimulated=3.9	
Sekhon *et al*., 2017	Cumulative gonadotropin dose used, duration of stimulation	NA	47% no association of cumulative dose and aneuploidy (adjusted OR=1.0.49, *p*=0.232)	NA	NA	NA	
Labarta *et al*., 2017	Number of euploid embryos	Low=11.5 High=23.8	Low=39% High=639%, *p*=0.2	NA	Low=2.7 . High=5.0	NA	
Barash *et al*., 2017	Aneuploidy Rate per dosage and per age	16,3	<35 years old: 62.86% 35–37 years old: 55.75% 38-40 years old: 42.23% >41 years old: 27.53%	NA	NA	NA	
Venetis *et al*., 2019	Number of euploid embryos	11.7 (11.1–12.2)	Euploidy 17.4% (15.5–19.3), Aneuploidy 82.6%	NA	1.0 (0.9–1.1)	NA	
Thorne *et al*., 2019	Aneuploidy Rate	hCG=11.5 aGnRH=19.3	hCG=30.3% *vs* . aGnRH=37.8% MLRA: no differences between groups	NA	NA	NA	

*RCT: randomized controlled trial; IU: international units; USA: United States of America; FSH: follicle stimulating hormone; LH: luteinizing hormone; hMG: human menopausal gonadotropins; NA: not applicable; GnRH: gonadotropin-releasing hormone; rhCG: recombinant human chorionic gonadotropin; aGnRH: agonist of the GnRH; IVF: in vitro fertilization; FISH: Fluorescence in situ hybridization; RIF: repeated implantation failure; IUI: intrauterine insemination; POCs: products of conception; PGT: preimplantation genetic testing; array comparative genomic hybridization; qPCR-based CCS: quantitative polymerase chain reaction – based comprehensive chromosome screening; SNPs: single nucleotide polymorphism; NGS: next-generation sequencing; ITT: intention-to-treat; D3: cleavage-stage embryo.

### Principal Findings

#### Non-stimulated vs. Stimulated IVF

Three studies compared the incidence of aneuploidy in non-stimulated *vs*. stimulated cycles. First, Verpoest *et al*. (2008) prospectively followed 30 patients who underwent a natural cycle, of which 20 reached retrieval, 19 oocytes were successfully retrieved (0.6/patient), 15 MIIs fertilized and developed into 11 embryos which can biopsied: 4 aneuploid, 6 euploid (0.2/patient) and 1 undiagnosed. There was no control group. Aneuploidy rate, performed by FISH analysis, was 36.4% (Verpoest *et al*., 2008). An ITT analysis yielded 20% (6/30) of the patients with an euploid embryo.

Similarly, Massie *et al*. (2011) analyzed the POC of missed abortions from infertility patients who conceived naturally (n=50) versus infertility patients who conceived under FSH stimulation (n=179). Despite similar age in both groups, the authors reported aneuploidy in POCs in 70% of the samples when patients conceived naturally, *vs*. 63% after FSH stimulation for IVF (Massie *et al*., 2011). Although this study provides an indirect comparison, it shows that patients without stimulation are at similar risk of generating abnormal embryos.

Lastly, Labarta *et al*. (2012) performed a prospective cross-over study in which the same donor underwent an unstimulated and a stimulated COS cycle. The aneuploidy rate reported was 35.3% (18/51) in the unstimulated group and 40.6% (123/303) in the stimulated group. In terms of euploid embryos per patient, there was 1.8 in the unstimulated group *vs*. 3.9 in the stimulated group. Of note, the stimulated group included 47 patients and 97.9% reached the biopsy stage. Conversely, in the unstimulated group, 185 donors were enrolled and only 27.6% (n=51) reaching biopsy [65.4% (n=121) reached retrieval, 53.5% (n=99) had an oocyte retrieved; 48.1% (n=89) had an MII retrieved; 30.8% (n=57) fertilized] (Labarta *et al*., 2012). Considering that almost all arrested embryos are not euploid (Qi *et al*., 2014), the proportion of included patients who achieved an euploid embryo was actually 58.6% (180/307) in the stimulated group *vs*. 16.2% (30/185) in the unstimulated group. Therefore, the actual aneuploidy rate in the unstimulated cycle was 76.8% (76/99 oocyte retrievals) *vs*. 40.6% (123/303) in the stimulated cycle. Correspondingly, the true number of euploid embryos per patient of the unstimulated was 1.0 *vs*. 3.9 in the stimulated group.

In summary, the absence of stimulation does not preclude embryonic aneuploidy. Special attention should be given when comparing patients, because most of the times patients that do not reach biopsy stage are not taken into account in the final analysis.

#### Low vs. Conventional Gonadotropin Dose

Two studies have compared a conventional *vs*. a reduced dose. The first study was published by Baart *et al*. (2007), and it is the only RCT included in this systematic review. The authors compared 111 patients randomized to a conventional (n=44) versus reduced (n=67) protocol with PGT under FISH analysis. Interestingly, the authors terminated the study prematurely due to a significantly lower embryo aneuploidy rate per patient observed after a reduced dose (Baart *et al*., 2007). There are two key comments to this manuscript: first, the authors reported that conventional stimulation was associated with a 38% euploidy rate (61/159), versus 50% euploidy rate (71/143) with the reduced dose (*p*=0.02). The authors stated that the reason for interruption was that we met a Pocock critical bound (the *p*-value was <0.0354). Of note, the p-value for that comparison is 0.048. Also, when analyzing the tables included, the aneuploidy rate was actually 26% (41/159) with conventional stimulation, versus 22% (31/143) with a reduced dose, with a *p*-value of 0.4. Second and most importantly, if the authors would have done an ITT analysis, the results would have been more valid and practical for advising patients. The authors assert that both groups had 1.8 euploid embryos available for transfer. If all patients would have been considered, patients undergoing conventional stimulation would have had 2.5 euploid embryos and patients undergoing mild stimulation would have had 1.4 euploid embryos (table II of Baart *et al*.'s manuscript, divided into 44 and 67 patients, respectively, instead of dividing the results in 33 and 40 patients, who reach biopsy stage, respectively) (Table 1).

Later, Rubio *et al*. (2010) published a prospective cross-over study including 32 donors who underwent a COS with standard dose (225 IU) and a subsequent cycle with a 30% dose reduction (150 IU). Twenty-two donors completed both cycles, with a 31.3% cancellation rate in the reduced-dose group. The authors found significant differences in euploidy rate: 22.5% (standard dose) *vs*. 16.1% (reduced dose). Additionally, the authors reported a statistical difference in the proportion of blastocysts per biopsied embryo (66.8%) (139/296) *vs*. 75.6% (115/152), *p*=0.04) in favor of the reduced dose. And in terms of clinical outcomes, the authors reported a pregnancy rate (PR) per transfer of 56.0% (14/25) with a standard dose *vs*. 52.2% (12/23) with a reduced dose (Rubio *et al*., 2010). Unfortunately, satisfactory conclusions cannot be drawn because the authors also did not perform an ITT analysis. Considering all patients assigned per arm (included the 10 patients in the reduce dose who were canceled), the proportion of blastocysts per biopsied embryos would have been 69.3% (205/296) versus 75.6% (115/152), rendering a *p*>0.05. Additionally, if we take into account the biopsied embryos (n=82) from the 10 patients under conventional-dose, there are an additional 33 euploid embryos, which translates into 3.1 euploid embryos available per donor in the standard dose *vs*. 1.8 in the reduced-dose (instead of 3.1 *vs*. 2.7). And most importantly, PR per transfer would have been 63.9% (23/36) in the standard-dose group *vs*. 52.2% (12/23) in the reduced-dose group (Rubio *et al*., 2010).

Although both studies conclude that a reduced dose is associated to a lower proportion of aneuploidy, it is not clear that, when including all patients in the analysis, a reduced dose would provide a higher euploidy rate. Studies with an ITT design are needed to truly determine a strategy's aneuploidy rate and total number of embryos biopsied.

#### GnRH Agonist versus GnRH Antagonist

Only one study compared the incidence of aneuploidy correlated to the type of pituitary suppression. Kyrou *et al*. (2011) compared 694 consecutive cycles in patients ≤37 years undergoing a long-agonist (n=320) *vs*. antagonist protocol (n=374 cycles) under the same COS criteria. The Patients' embryos underwent D3 biopsy, FISH analysis and D5 embryo transfer (ET). The aneuploidy rate was statistically similar between the embryos of the two study groups (49.9±28.1 *vs*. 50.2±26.6; *p*=0.922). A multivariate linear regression analysis confirmed that the total abnormality ratio was not influenced by the type of stimulation when simultaneously adjusting for age, rank of trials, indication for PGT, total gonadotropin amount, number of COCs, and number of 2PN embryos (Kyrou *et al*., 2011).

Unfortunately, the authors only included patients who reached biopsy; thus, an ITT analysis could not be performed. The authors concluded that there was no difference in the proportion of aneuploidy when using an agonist or antagonist protocol (Kyrou *et al*., 2011).

#### FSH-Only versus LH-Containing Gonadotropins

Two studies have weighed the impact of LH-containing gonadotropins on the chromosomal status of preimplantation embryos during IVF (Weghofer *et al*., 2008; 2009). First, Weghofer *et al*. (2008) studied the effect of COS with FSH-only medications (n=52) *vs*. human menopausal gonadotropin (hMG) (n=52) in patients undergoing a long agonist protocol. Overall, the rate of euploidy, calculated on a per-patient basis, was significantly higher in patients under hMG (45.3% versus 69.8%, respectively, *p*<0.01). Interestingly, the total number of euploid embryos was similar (FSH 3.1 *vs*. hMG 3.3). This difference is explained because patients who utilized rFSH produced significantly more aneuploid and complex abnormal embryos than patients undergoing hMG stimulation (rFSH: aneuploid embryos 2.2, complex abnormal embryos 2.9; hMG aneuploid embryos 0.9, complex abnormal embryos 1.2, *p*<0.01). Therefore, the proportion was in favor of the hMG group. A linear multi-regression analysis (age, IVF attempts, diagnosis, type and dosage of gonadotropins used, oocytes retrieved, usable embryos) confirmed that FSH-only stimulation influenced aneuploidy rates (*p*<0.02) (Weghofer *et al*., 2008).

The same group evaluated patients undergoing IVF under an antagonist protocol (Weghofer *et al*., 2009). Both groups produced statistically similar numbers of euploid embryos (FSH, 2.1 *vs*. FSH/hMG, 1.9) and similar euploidy rates, calculated on a per-patient basis (FSH: 29.4% *vs*. FSH/hMG: 25.7%). After a linear multi-regression analyses, ploidy status was not impacted by the gonadotropin used (FSH *vs*. FSH/hMG), the indication for PGD (after correction for age), nor the duration of GnRH antagonist.

Consequently, with these results and given that during an antagonist cycle LH is not suppressed at the beginning of the cycle, we can infer that initial gonadotropin-dependent follicle selection is maintained during antagonist cycles, whereas it might be in agonist cycles, in which endogenous LH concentrations are reduced very in the cycle.

#### Triggering of final oocyte maturation: r-hCG vs. GnRH-agonist

One recent study evaluated euploidy rates according to trigger medication. Thorne *et al*. (2019) compared 366 patients involving 539 cycles (rhCG=336 *vs*. aGnRH=203). Raw analysis showed that the euploidy rate was higher when aGnRH trigger was used (37.8%±2.1% *vs*. 30.3%±1.8%, *p*=0.01). After a multivariate linear regression analysis, trigger medication was no longer predictive of ploidy status [B:-0.02, 95% CI (-0.08, 0.05), F (0.19,1), *p*=0.67] (Thorne *et al*., 2019), with age being the only predictive factor. Of note, in everyday practice, a GnRH trigger is most likely used in younger, hyper-responsive patients, which could explain the differences in baseline characteristics (younger, higher AMH, lower BMI, lower total gonadotropin dose, higher peak estradiol level).

#### According to Ovarian Response

Seven non-randomized studies have analyzed whether the aneuploidy rate was associated with the total number of oocytes or embryos generated (Haaf *et al*., 2009; Rubio *et al*., 2010; Ata *et al*., 2012; Barash *et al*., 2017; Labarta *et al*., 2017; Sekhon *et al*., 2017; Venetis *et al*., 2019).

The first study correlating ovarian response and aneuploidy was performed in Germany (Haaf *et al*., 2009), and given that German law does not allow PGT on blastomeres, the authors performed polar body analysis with FISH, and analyzed the aneuploidy rate per cohort size (1-5,6-10, >10 oocytes retrieved) and per age (<35, 35-40, >40 years old). The authors reported that aneuploidy rate increased with increasing age. Interestingly, the aneuploidy rate was similar in each age group and increasing oocyte yield, except in patients < 35years, in which the aneuploidy rate increased with increasing number of oocytes: 1-5 oocytes 23.3%; 6-10 oocytes 34.9%; >10 oocytes 50.9%. Although the authors claim that the increased chromosome error rate may be explained by a non-physiological ovarian stimulation (which interferes with chromosome segregation behavior during maternal meiosis) and that lower oocyte yields may represent a more appropriate response to ovarian stimulation (allowing only the most competent follicles and oocytes to develop and, therefore, reduce oocyte error rates) is important to consider that, when analyzing only patients that reach the biopsy stage or had at least one embryo biopsied, by definition we are excluding patients who are at the higher risk of producing an embryonic aneuploidy: poor/low responder patients who are generally older or with low quality oocytes and/or embryos. With these in mind, most of the patients who would have had between 1-5 oocytes retrieved with an abnormal oocyte did not reach biopsy and analysis.

Rubio *et al*. (2010) performed a sub-analysis comparing patients who after two cycles, first under 225UI then a second cycle under 150UI, responded with similarly number of oocytes retrieved. Although the authors found higher fertilization rates (82.3% *vs*. 71.4%) with the reduced dose, the proportion of euploid blastocysts per MII retrieved (11.4% *vs*. 20.2%, *p*>0.05) and the mean euploid blastocysts per donor (1.5 *vs*. 1.5, *p*>0.05) were similar in both groups (Rubio *et al*., 2010).

Next, Ata *et al*. (2012) ran a retrospective analysis under aCGH including 1218 blastocysts from 203 women, with results stratified by age and number of embryos generated. The euploidy rates were comparable within each age group (<35, 35-39, 40-42, ≥43), but decreased with increasing age: 1-4 blastocysts: 70.2%, 66.0%, 49.1%, 34.2%, 16.7%, respectively; 5-7 blastocysts: 77.5%, 69.9%, 52.3%, 31.0%, 13.3%, respectively; 8-10 blastocysts: 62.4%, 56.7%, 48.3%, 27.4% 22.5%, respectively; and >10 blastocysts 66.7%, 53.3%, 51.4%, 40.9%, 16.7%, respectively (Ata *et al*., 2012). A linear regression analysis showed that for every 1-year increase in age, the euploidy rate decreased 2.9%, and a logistic regression analysis revealed that the odds of having at least one euploid embryo was significantly decreased by female age: OR 0.82 (95% CI 0.70-0.94, *p*=0.006), interpreted as 28% less likely with each extra year. Nevertheless, the odds of having at least one euploid embryo increase by every additional embryo available: OR 1.55(95% CI 1.25-1.93), *p*<0.001, interpreted as 1.5 times more likely with each extra embryo (Ata *et al*., 2012).

Subsequently, Sekhon *et al*. (2017) ran a retrospective study using a logistic regression fit with generalized estimating equations (GEE) for large data analysis, stratifying patients by treatment protocol, duration of stimulation, and by total gonadotropin dose. After controlling for several factors (age, FSH, follicle count, protocol, days of stimulation protocol, and diagnosis, there was no significant association between cumulative dose of gonadotropins and the likelihood of aneuploidy (adjusted OR 1.049, *p*=0.232). Nevertheless, if more than 12 days of stimulation were necessary, a 20% increase in the likelihood of aneuploidy was found (adjusted OR 1.20, 95% CI 1.125-1.282, *p*<0.001), with an extra 16.4% increase in the odds of aneuploidy with every 1000 UI increase (adjusted OR 1.164, *p*=0.002) (Sekhon *et al*., 2017). These data could suggest a susceptibility to low oocyte quality, which is often seen in ageing patients and/or patients with low ovarian reserve.

Subsequently, Labarta *et al*. (2017) performed a post-hoc analysis of a prospective cohort study from 2012 (Labarta *et al*., 2012). The authors reported that when the number of oocytes obtained was ≥17 oocytes, the mean number of euploid embryos was 5.0, while it was 2.6 when the ovarian response was lower (*p*<0.001). The authors found that the number of euploid embryos was inversely related to the ovarian sensitivity index, a correlation of the amount of gonadotropins required per oocyte retrieved (Huber *et al*., 2013), reflecting better ovarian competence. The authors concluded that a high ovarian response through moderate doses of gonadotropin in young normo-ovulatory women does not increase the rate of embryonic aneuploidy (Labarta *et al*., 2017).

Barash *et al*. (2017) analyzed whether different doses of gonadotropins influence euploidy rates. The authors performed a retrospective study of SNPs/PGT outcome data on blastocysts biopsied on D5/D6, from a total of 4034 embryos analyzed. The authors conducted a retrospective study of 4034 embryos on D5/D6 analyzed by SNPs. The cycles were analyzed by total gonadotropin dose (<3000 IU, 3000-5000 IU and> 5000 IU), by retrieved oocytes (1-5, 5-10, 10-15 and> 15 oocytes) and by patient age (<35, 35-37, 38-40 and ≥41 years). Euploidy rates within the same age group were reported to be similar, regardless of total dose used and/or oocytes retrieved. Furthermore, the euploidy rates were not affected by high doses of gonadotropin (450 IU per day) in patients <38 years. In the group of patients ≥38 years, the authors found a gradual decrease in euploidy rates when high doses were used, although it only reached statistical significance in patients aged 38 to 40 years (Barash *et al*., 2017). Interestingly, within each age group, the usable blastocyst rate was similar when <3000 IU gonadotropins were used in cycles where more than 5000 IU were used. The authors observed a difference in usable blastocyst rate, with increasing age, although it only reached statistical significance in patients aged 35 to 37 years (OR=1.417, 95% CI 1.047-1.918, *p*=0.024).

Lastly, Venetis *et al*. (2019) analyzed 724 patients using D3 biopsy and aCGH and included all significant population and stimulation characteristics as covariates in a multivariate regression GEE, as well as an interaction term between female age and number of oocytes, revealed that female age (coefficient: −0.06, 95% CI −0.11 to −0.016, *p*=0.009) and the number of oocytes retrieved (coefficient: +0.40; 95% CI 0.24-0.56, *p*<0.001) remain the only significant prognosticators of the total number of euploid embryos.

Among the studies associating ovarian response to aneuploidy, only Haaf *et al*. (2009) reported an increased aneuploidy rate, although there were important statistical limitations. Overall, all other 6 studies reported similar results, concluding that ovarian response did not alter euploidy rate or the total number of euploid embryos. Moreover, a case can be made towards better results with increasing number of biopsied embryos.

## DISCUSSION

This systematic review investigated a topic of great importance for reproductive endocrinologists in the field of reproductive medicine, and, to our knowledge, it is the first of its kind. Concerning the impact of COS on chromosome imbalance in human embryos, the current review did not find a clear correlation. On one hand, it has been previously proved that natural cycles are also associated with aneuploidy, but on the other hand, it seems that more intensive stimulations are indeed associated to a higher proportion of aneuploidy. Nevertheless, given that a higher response is associated with more oocytes retrieved, this translates into potentially more euploid embryos available for transfer.

Up to the present time, the only factor incontrovertibly linked to human aneuploidy is increasing maternal age (Franasiak *et al*., 2014). Even though chromosomal errors seem to be linked to a generalized oocyte disorganization rather than to a single/specific oocyte alteration, there is increasing evidence supporting the fact that each aneuploidy event is not dependent on a specific variable, but on a group of variables. To date, it has been impossible to isolate if the gonadotropin dosage actually distresses the oocyte or if the aneuploidy is simply a reflection of its low quality. If, for a moment, we assume that COS does, in fact, impact embryonic aneuploidy, what would it be preferable to have, a lower ratio of aneuploidy or higher total number of euploid embryos? Morin *et al*. (2014) published that in patients with ≤3 euploid blastocysts, clinical PR was higher in double, compared to single embryo transfers; however, in patients with ≥4 euploid blastocysts, clinical PR was not decreased with single embryo transfer. These findings support the hypothesis that euploid embryo cohort size is an important prognosis factor in ART treatment, and the number of euploid embryos has to be taken into account, more significantly than the ratio of euploid/aneuploid embryos (Morin *et al*., 2014; Labarta *et al*., 2017).

One particular strength is the detailed analysis on a per study basis and grouping studies with similar intervention criteria. Secondly, whenever possible, the authors ran an ITT analysis, with the purpose of homogenizing the denominator in the analysis groups

Limitations of the present systematic review should be acknowledged. First, only one RCT has been performed. Even though the quality of the rest of the studies is high (NOS above 7), bias associated with retrospective studies cannot be ruled out. Second, the studies included presented a large diversity in the assessment of embryonic aneuploidy, and, given the great heterogeneity between studies, a meta-analysis could not be performed. Important differences consisted in the different platforms used to analyze aneuploidy and the embryo stage at which the biopsy was performed. Third, in those studies correlating ovarian response with aneuploidy, no information regarding the number of aneuploid embryos and the number of embryos biopsied was reported, which prevented pooling the data. In this regard, an effort was made to contact corresponding authors, but it was not possible to obtain the full data. And fourth, this review did not address the problem of mosaicism (Munné *et al*., 2002; Cram *et al*., 2019). For the purpose of this review, all embryonic abnormalities were considered as aneuploidy, mainly because that is how abnormalities were reported in the studies included; therefore, mosaicism was included as an aneuploid embryo when reported. Recently, the genomic technologies available have improved in sensitivity and resolution and have permitted a more complete spectrum of chromosome abnormalities to be identified, but long-term studies are needed to understand the clinical impact of mosaicism. In our opinion, whether mosaicism is considered separately or together with aneuploidy, does not changes the analysis, as it is still considered to be an abnormal result and associated to lower clinical outcomes (Munné *et al*., 2020).

Remarkably, no large randomized clinical trial on this topic has yet been conducted. Further evidence is needed to ascertain if there is a negative impact of COS, especially at the cellular level. Furthermore, biomarkers to predict minimal effective dose to achieve the highest reproductive potential are needed.

## Appendix

**Supplemental Table 1.** Search Builder according to Patients, Intervention, Comparison and Outcomes (PICO) model.

PATIENTS		INTERVENTION		OUTCOME		COMPARISON
“Ovulation Induction” OR “ovarian stimulation” OR COS OR “ovarian hyperstimulation” OR COH	AND	“Preimplantation Diagnosis” OR “preimplantation genetic screening” OR PGS OR “preimplantation genetic testing” OR PGT OR “preimplantation genetic diagnosis” OR PGD OR “genetic screening” OR “genetic testing” OR “comprehensive chromosome screening” OR “comprehensive chromosome analysis” OR “next-generation sequencing” OR NGS OR “comparative genomic hybridization” OR CGH OR “array comparative genomic hybridization” OR aCGH OR “single nucleotide polymorphism” OR “Polymorphism, Single Nucleotide” OR SNP OR “polymerase chain reaction” OR PCR OR “quantitative polymerase chain reaction” OR qPCR OR “whole genome sequencing” OR “microarray analysis”	AND	Aneuploidy OR aneuploid* OR euploidy OR euploid* OR “chromosome disorders” OR “chromosome aberrations/chemically induced”	AND	“Fertilization *in Vitro* ” OR “ *in vitro* fertilization” OR IVF OR “Reproductive Techniques, Assisted”

**Supplemental Table 2.** Characteristics and Newcastle–Ottawa scale score of studies included in a systematic review of the association of ovarian stimulation with embryonic aneuploidy in in vitro fertilization cycles coupled with for preimplantation genetic testing.

Author	Selection				Comparability	Outcome			Quality Score
	Representativeness Quality Score of Exposed Cohort	Selection of the Non-Exposed Cohort from Same Source as Exposed Cohort	Ascertainment of Exposure	Outcome of Interest Was Not Present at Start of Study	Comparability of Cohorts	Assessment of Outcome	Follow-Up Long Enough for Outcometo Occur	Adequacy of Follow-Up	
Baart et al., 2007	*	*	*	*	**	*	*		8
Weghofer *et al*., 2008	*	*	*	*	**	*	*	*	9
Verpoest *et al*., 2008	*		*	*		*	*		5
Haaf *et al*., 2009			*	*	**		*	*	6
Weghofer *et al*., 2009	*	*	*	*	**	*	*	*	9
Rubio *et al*., 2010	*	*	*	*	**	*	*		8
Kyrou *et al*., 2011	*	*	*	*	**	*	*	*	9
Massie *et al*., 2011	*	*	*	*		*	*	*	7
Ata *et al.*, 2012	*	*	*	*	**	*	*	*	9
Labarta et al., 2012	*	*	*	*	**	*	*		8
Sekhon *et al*., 2017	*	*	*	*	**	*	*	*	9
Labarta *et al*., 2017	*	*	*	*	**	*	*	*	9
Barash *et al*., 2017	*	*	*	*	**	*	*	*	9
Venetis *et al*., 2019	*	*	*	*	**	*	*	*	9
Thorne *et al*., 2019	*	*	*	*	**	*	*	*	9

* Based on the Newcastle–Ottawa scale. A study can be awarded a maximum of one star for each item within the Selection and Exposure categories. A maximum of two stars can be given for Comparability. Evidence was considered low (0–4), moderate (5–6) or high quality (7–9).
